# Zosuquidar Promotes Antitumor Immunity by Inducing Autophagic Degradation of PD‐L1

**DOI:** 10.1002/advs.202400340

**Published:** 2024-09-04

**Authors:** Ling Ding, Hongjie Guo, Jie Zhang, Mingming Zheng, Wenjie Zhang, Longsheng Wang, Qianqian Du, Chen Zhou, Yanjun Xu, Honghai Wu, Qiaojun He, Bo Yang

**Affiliations:** ^1^ Zhejiang Province Key Laboratory of Anti‐Cancer Drug Research Institute of Pharmacology and Toxicology College of Pharmaceutical Sciences Zhejiang University Hangzhou 310058 China; ^2^ Nanhu Brain‐Computer Interface Institute Hangzhou 311100 China; ^3^ The Cancer Hospital of University of Chinese Academy of Sciences (Zhejiang Cancer Hospital) Chinese Academy of Sciences Hangzhou 310022 China; ^4^ The Innovation Institute for Artificial Intelligence in Medicine Zhejiang University Hangzhou 310018 China; ^5^ Cancer Center of Zhejiang University Hangzhou 310058 China; ^6^ School of Medicine Hangzhou City University Hangzhou 310015 China

**Keywords:** ABCB1, autophagy, ER retention, PD‐L1, zosuquidar

## Abstract

The intracellular distribution and transportation process are essential for maintaining PD‐L1 (programmed death‐ligand 1) expression, and intervening in this cellular process may provide promising therapeutic strategies. Here, through a cell‐based high content screening, it is found that the ABCB1 (ATP binding cassette subfamily B member 1) modulator zosuquidar dramatically suppresses PD‐L1 expression by triggering its autophagic degradation. Mechanistically, ABCB1 interacts with PD‐L1 and impairs COP II‐mediated PD‐L1 transport from ER (endoplasmic reticulum) to Golgi apparatus. The treatment of zosuquidar enhances ABCB1‐PD‐L1 interaction and leads the ER retention of PD‐L1, which is subsequently degraded in the SQSTM1‐dependent selective autophagy pathway. In CT26 mouse model and a humanized xenograft mouse model, zosuquidar significantly suppresses tumor growth and accompanies by increased infiltration of cytotoxic T cells. In summary, this study indicates that ABCB1 serves as a negative regulator of PD‐L1, and zosuquidar may act as a potential immunotherapy agent by triggering PD‐L1 degradation in the early secretory pathway.

## Graphical abstract

Newly synthesized programmed death‐ligand 1 (PD‐L1) needs to be sequentially transported to endoplasmic reticulum (ER) and Golgi apparatus for post‐translational modification. The adenosine 5'‐triphosphate (ATP) binding cassette subfamily B member 1 (ABCB1) modulator zosuquidar disrupts PD‐L1 translocating from ER to Golgi apparatus, dramatically triggers autophagic degradation of PD‐L1, and exhibits significant anti‐tumor effect in vivo.

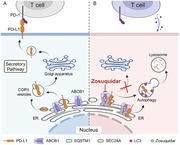

## 1 Introduction

PD‐L1 (programmed death‐ligand 1) expressed on tumor cell surface is frequently upregulated to evade immune surveillance by interacting with PD‐1 (programmed cell death protein 1) expressed on T cells.^[^ ^1^ ^]^ Monoclonal antibodies targeting PD‐1‐PD‐L1 axis have demonstrated significant clinic benefits in multiple types of solid tumors, while disadvantages such as immune‐related adverse effects (irAEs), intrinsic or acquired resistance, and way of intravenous administration, severely limited the clinical application of PD‐1‐PD‐L1 antibodies.^[^ ^2^, ^3^, ^4^, ^5^ ^]^ Accumulated evidence has highlighted the opportunity to develop small‐molecule agents that modulate PD‐L1 expression based on regulatory mechanisms.^[^ ^6^, ^7^ ^]^ Compounds such as JQ1, eFT508, and curcumin suppressed tumor growth in vivo by reducing PD‐L1 expression at the transcriptional, translational, and post‐translational levels, respectively.^[^ ^8^, ^9^, ^10^ ^]^ Verticillin A was also shown to reduce PD‐L1 abundance by inhibiting MLL1‐mediated H3K4me3 at the *CD274* promoter.^[^ ^11^ ^]^ A serious of kinases inhibitors including wortmannin (phosphoinositide 3‐kinase inhibitor), tofacitinib (Janus kinase inhibitor), as well as rapamycin (mammalian target of rapamycin inhibitor) also exhibited significant inhibitory effects on PD‐L1 expression, and have been studied in preclinical or clinical trials.^[^ ^7^ ^]^ Therefore, the development of small‐molecule agents based on PD‐L1 expression regulation may provide promising strategies for immunotherapy.^[^ ^12^, ^13^, ^14^ ^]^

Recent studies have emphasized the crucial role of intracellular transport process in PD‐L1 expression and immunosuppressive function. The expression of CMTM6 (CKLF like MARVEL transmembrane domain containing 6) and SGLT2 (solute carrier family 5 member 2) has been found to stabilize PD‐L1 by inducing internalized PD‐L1 recycle back to the cell membrane.^[^ ^15^, ^16^ ^]^ Both CMTM6 depletion and pharmacological inhibition of SGLT2 by canagliflozin exhibited significant anti‐tumor efficiency through triggering PD‐L1 degradation. In addition, calpain was shown to induce recycling endosomes (RE) loaded with PD‐L1 proteins to transfer to the plasma membrane.^[^ ^17^ ^]^ By inhibiting calcium flux, amlodipine triggering autophagic degradation of PD‐L1 accumulated on RE, thus increasing the infiltration of CD8^+^ T cells in tumor tissues. Moreover, inducing the redistribution of PD‐L1 to mitochondria was also beneficial for overcoming chemoimmunotherapy resistance in triple‐negative breast cancer.^[^ ^18^ ^]^ Furthermore, inhibiting PD‐L1 trafficking from endoplasmic reticulum (ER) to Golgi apparatus also showed sufficient tumor suppression effect. Several compounds such as Metformin and BMS1166 disrupted the N‐glycosylation modification of PD‐L1 and thereby prevented its ER export and triggered endoplasmic reticulum‐associated degradation.^[^ ^19^, ^20^ ^]^ Depletion of THADA (thyroid adenoma associated gene), which is involved in PD‐L1 ER to Golgi trafficking, induced ER retention and degradation of PD‐L1.^[^ ^21^ ^]^ Therefore, exploring the regulatory mechanism and critical regulators of PD‐L1 intracellular distribution would be beneficial for the development of targeted PD‐L1 small molecule inhibitors.

In the present study, we found that zosuquidar, a selective modulator of ABCB1 (ATP binding cassette subfamily B member 1), dramatically reduced PD‐L1 expression through the cell‐based high‐content screening. We also showed that zosuquidar disrupted PD‐L1 translocating form ER to Golgi apparatus in a target‐dependent manner, which led the ER retention of PD‐L1 and then triggered its SQSTM1 (sequestosome 1)‐dependent selective autophagy. In CT26 mouse model and a humanized xenograft mouse model, the treatment of zosuquidar significantly reduced the expression of PD‐L1 and suppressed tumor growth by inducing the infiltration of cytotoxic T cell. Together, our work identified zosuquidar as a PD‐L1 degrader by inducing PD‐L1 autophagic degradation in the early secretory pathway and may act as a potential immunotherapy drug.

## 2 Result

### 2.1 Zosuquidar Suppressed PD‐L1 Expression In Vitro and In Vivo

**Figure 1:**
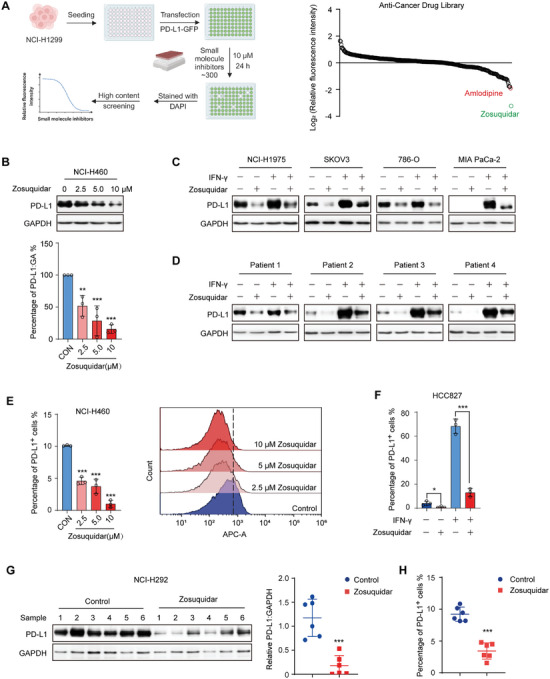
Zosuquidar downregulated PD‐L1 expression. A) Schematic illustration of cell‐based high content screening (left) and the effect of over 300 anti‐cancer drugs on GFP fluorescence intensity (right). NCI‐H1299 cells were transfected with PD‐L1‐GFP plasmid and then treated with anti‐cancer drug library (10 × 10^−6^ m) for 24 h. Fluorescence intensities were recorded and analyzed by CellReporterXpress module in ImageXpress Pico system. Amlodipine was used as a positive control which has been reported to trigger PD‐L1 degradation. B) Representative western blot (top) of PD‐L1 expression in NCI‐H460 cells treated with different concentrations (2.5 × 10^−6^, 5 × 10^−6^, and 10 × 10^−6^ m) of zosuqudiar for 24 h, and quantified using Image J grayscale analysis (bottom). C,D) Western blot of PD‐L1 expression in NCI‐H1975 (NSCLC), SKOV3 (ovarian cancer), 786‐O (renal carcinoma), Mia PaCa‐2 (pancreatic cancer), and 4 human primary patient‐derived lung cancer cells with the treatment of zosuquidar (10 × 10^−6^ m) alone or together with IFN‐γ (10 ng mL^−1^) for 24 h. E,F) Flow cytometry analysis of PD‐L1 expression on the cell surface in NCI‐H460 cells treated with different concentrations (2.5 × 10^−6^, 5 × 10^−6^, and 10 × 10^−6^ m) of zosuqudiar for 24 h, and HCC827 cells with the treatment of zosuquidar (10 × 10^−6^ m) alone or together with IFN‐γ (10 ng mL^−1^) for 24 h. G) Western blot and H) flow cytometry analysis of PD‐L1 expression in vivo experiment, and the western blot data was quantified using Image J grayscale analysis. BALB/c nude mice bearing NCI‐H292 cells were treated with vehicle or zosuquidar (90 mg kg^−1^, intragastric administration) daily for 10 d (*n* = 6). Data were presented as the mean ± SD of triplicate independent experiments. Unpaired two‐tailed Student's *t*‐test (two groups) and one‐way ANOVA with Dunnett's post hoc test (more than two groups) were used to determine statistical significance of experimental data. ***, *P* < 0.001; **, *P* < 0.01; *, *P* < 0.05.

To identify small‐molecule inhibitors that can suppress PD‐L1 expression, we performed cell‐based high‐content screening in non‐small‐cell lung cancer (NSCLC) cell line NCI‐H1299 and over 300 anti‐cancer drugs were evaluated in our screening (Table S1, Supporting Information). To test and verify our experimental system, amlodipine, which has been reported to promote PD‐L1 degradation, was used as the positive control.^[^ ^17^ ^]^ As shown in **Figure** **1**A, amlodipine significantly reduced the fluorescence intensity as expected, and we noticed that zosuquidar, a selective modulator of ABCB1, showed the most inhibitory effect among these drugs. To further validate this result, we treated NCI‐H460 cells with different concentrations of zosuquidar, and the expression of PD‐L1 was down‐regulated by zosuquidar in a dose‐dependent manner (Figure 1B). Considering the expression of PD‐L1 is frequently induced by Interferon gamma (IFN‐γ) in vivo, we evaluated the impact of zosuquidar on PD‐L1 expression under both basal and IFN‐γ‐induced conditions. Consistently, zosuquidar reduced PD‐L1 expression under both basal and inducible conditions in NSCLC (NCI‐H1975), ovarian cancer (SKOV3), renal carcinoma (786‐O), and pancreatic cancer (MIA PaCa‐2) (Figure 1C). Similar results were also demonstrated in 4 primary patient‐derived lung cancer cells (Figure 1D). Since PD‐L1‐mediated immune escape mainly relies on its expression on the plasma membrane, we used flow cytometry analysis and found that zosuquidar also reduced the expression of cell‐surface PD‐L1 (Figure S1, Supporting Information and Figure 1E,F). In order to examine whether zosuquidar can down‐regulate PD‐L1 expression in vivo condition, the NSCLC cell line NCI‐H292 was subcutaneously inoculated into nude mice and zosuquidar (90 mg kg^−1^) was orally administered daily for 10 d. As shown in Figure 1G,H, the treatment of zosuquidar remarkably decreased the expression of PD‐L1 in tumors. Taken together, these results demonstrated that zosuquidar can suppress PD‐L1 expression in vitro and in vivo.

### 2.2 Zosuquidar Suppressed Tumor Growth in CT26 Mouse Model

**Figure 2:**
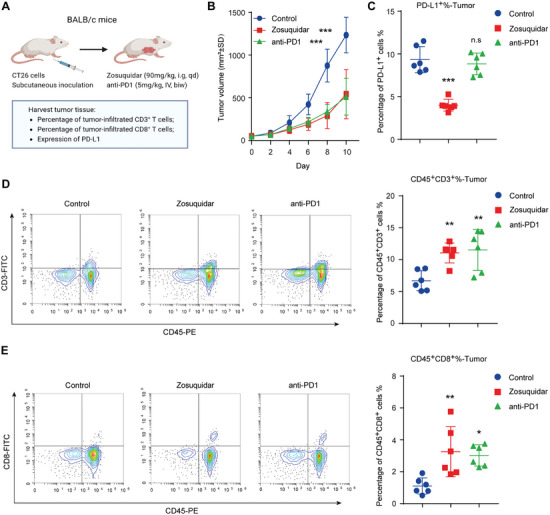
Zosuquidar inhibited tumor growth in syngeneic mouse model. A) Schematic illustration of CT26 mouse model. BALB/c mice were subcutaneously injected with CT26 cells and treated with zosuquidar (90 mg kg^−1^, intragastric administration) daily or anti‐PD‐1 (5 mg kg^−1^, intravenous injection) twice a week for 10 d (*n* = 6). B) Tumor volume of each group was measured every 2 d with a caliper. C) Cell‐surface expression of PD‐L1 in each tumor tissue was determined by flow cytometry. Tumor infiltrating D) CD45^+^CD3^+^ T cells and E) CD45^+^CD8^+^ T cells were detected by flow cytometry. Representative flow cytometry results were shown on the left. Data were presented as the mean ± SD. One‐way ANOVA with Dunnett's post hoc test was used to determine statistical significance of experimental data. ***, *P* < 0.001; **, *P* < 0.01; *, *P* < 0.05; n.s, not significantly different.

Since the expression of PD‐L1 on tumor cells severely impairs T cell‐mediated immunosurveillance by binding to PD‐1, we speculated whether zosuquidar could suppress tumor growth in vivo by reducing PD‐L1 expression. The in vitro study showed that zosuquidar can significantly suppress mPD‐L1 (mouse PD‐L1) expression in CT26 cell line (Figure S2A,B, Supporting Information). Therefore, we used the CT26 mouse model to evaluate the effect of zosuquidar on tumor growth, which is categorized as a highly immunogenic tumor model and widely used in PD‐1‐PD‐L1 related studies.^[^ ^22^, ^23^ ^]^ BALB/c mice bearing CT26 cells were treated with zosuquidar, PD‐1 monoclonal antibody (mAb), and control, respectively (**Figure** **2**A). We recorded tumor volume and mice body weight every 2 d, and the results indicated that zosuquidar exhibited obvious tumor growth‐inhibitive effects without affecting body weight, similar to PD‐1 mAb (Figure 2B and Figure S2C, Supporting Information). The expression of mPD‐L1 in tumor tissues was also decreased with the treatment of zosuquidar (Figure 2C). Moreover, zosuquidar significantly induced the infiltration of CD3^+^ T cells and CD8^+^ T cells in tumors (Figure 2D,E). The treatment of zosuquidar also increased animal survival (Figure S2D, Supporting Information). Taken together, zosuquidar reduced mPD‐L1 expression and exhibited anti‐tumor activity in the CT26 mouse model.

### 2.3 Zosuquidar Effectively Suppressed Tumor Growth in a PBMC‐Based Humanized Xenograft Model

**Figure 3:**
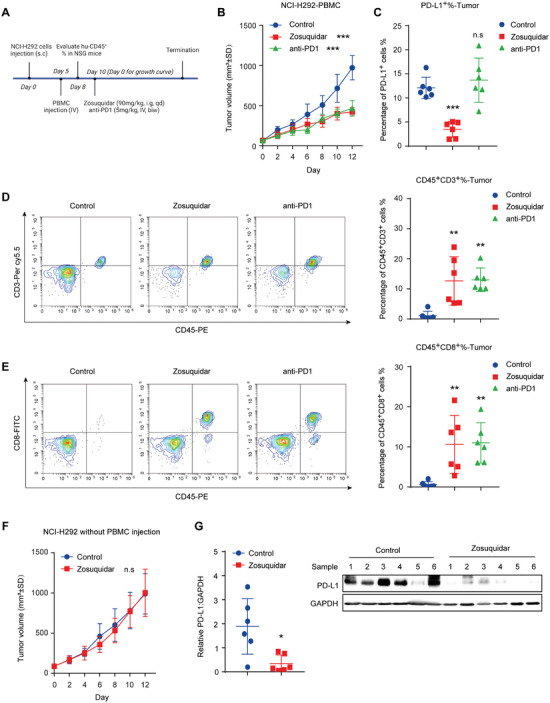
Zosuquidar suppressed tumor growth in a humanized immune‐transformation model. A) Schematic illustration of PBMC‐based humanized xenograft model. NSG mice bearing NCI‐H292 cells were intravenously injected with PBMCs and then treated with zosuquidar (90 mg kg^−1^, intragastric administration) daily or anti‐PD‐1 (5 mg kg^−1^, intravenous injection) twice a week for 12 d (*n* = 6). B) Tumor volume of each group was measured every 2 d in the PBMC‐based humanized xenograft model. C) The expression of PD‐L1 on the cell surface of each tumor tissues was determined by flow cytometry. Tumor infiltrating D) CD45^+^CD3^+^ T cells and E) CD45^+^CD8^+^ T cells were detected by flow cytometry. Representative flow cytometry results were shown on the left. (F) Tumor volume of each group in NSG mice without PBMC injection were measured every 2 d (*n* = 6). G) Western blot of PD‐L1 expression in tumor tissue after the treatment of vehicle or zosuquidar (90 mg kg^−1^, intragastric administration) for 12 d, and quantified using Image J grayscale analysis. Data were presented as the mean ± SD. Unpaired two‐tailed Student's *t*‐test (two groups) and one‐way ANOVA with Dunnett's post hoc test (more than two groups) were used to determine statistical significance of experimental data. ***, *P* < 0.001; **, *P* < 0.01; *, *P* < 0.05; n.s, not significantly different.

In recent years, humanized mouse model has become a reliable and representative tool for preclinical immuno‐oncology research.^[^ ^24^ ^]^ Therefore, we established a PBMC‐based humanized xenograft model to further evaluate the anti‐tumor efficacy of zosuquidar. After being subcutaneously inoculated with NCI‐H292 cells, human peripheral blood mononuclear cells (PBMCs) were intravenously injected into NSG mice to partially reconstitute the human immune system (**Figure** **3**A). Mice with Hu‐CD45^+^ T cells in peripheral blood were randomly divided into control group, zosuquidar group, and PD‐1 mAb group (Figure S3A, Supporting Information). Consistently, the treatment of zosuquidar or PD‐1 mAb significantly inhibited tumor growth without affecting body weight (Figure 3B and Figure S3B, Supporting Information). Flowcytometric analysis of tumor tissues showed that the expression of PD‐L1 was decreased in zosuquidar group, and the percentage of tumor‐infiltrated CD3^+^ and CD8^+^ T cells were induced with the treatment of zosuquidar or PD‐1 mAb (Figure 3C–E). To confirm whether the tumor inhibitory effect of zosuquidar was mainly relied on the existence of immune system, we further subcutaneously inoculated NCI‐H292 cells into NSG mice without PBMCs injection. Consistent with our hypothesis, zosuquidar did not exhibit any anti‐tumor effect, whereas the expression of PD‐L1 was downregulated (Figure 3F,G). Collectively, these data suggested that zosuquidar suppressed tumor growth by enhancing anti‐tumor immunity.

### 2.4 Zosuquidar Induced PD‐L1 Degradation via SQSTM1‐Dependent Selective Autophagy

**Figure 4:**
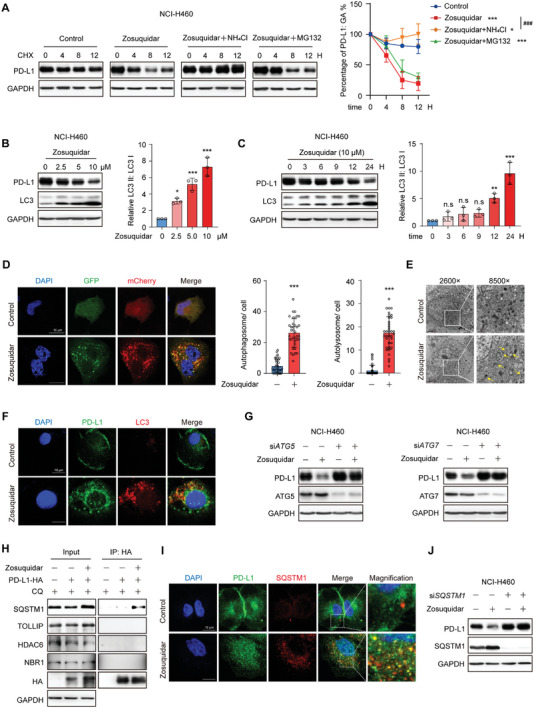
Zosuquidar induced PD‐L1 degradation through selective autophagy pathway. A) Representative western blot of PD‐L1 protein half‐life in NCI‐H460 cells treated with cycloheximide (10 µg mL^−1^) alone or cycloheximide plus zosuquidar (10 × 10^−6^ m) as well as MG132 (10 × 10^−6^ m) or NH_4_Cl (250 × 10^−6^ m) for indicated time. The quantification was shown on the right. B,C) The expression of LC3B in NCI‐H460 cells treated with different concentrations of B) zosuqudiar for 24 h or C) zosuqudiar (10 × 10^−6^ m) for 0, 3, 6, 9, 12, and 24 h. The quantification was shown on the right. D) Representative confocal fluorescence micrographs of NCI‐H460 cells transfected with mCherry‐GFP‐LC3B, followed by zosuqudiar treatment. Scale bar: 10 µm. The quantification of autophagosome and autolysosome puncta numbers was shown on the right. Control: *n* = 39; zosuquidar: *n* = 36. E) Transmission electron micrographs of NCI‐H460 cells with the treatment of zosuqudiar. For 2600× magnification, Scale bar: 5 µm; for 8500× magnification, Scale bar: 1 µm. Yellow arrowheads indicate autolysosomes. F) The colocalization of LC3 and PD‐L1 in NCI‐H460 cells after the treatment of zosuquidar was detected by immunofluorescence. Scale bar: 10 µm. G) Depletion of *ATG5* or *ATG7* influenced the effect of zosuquidar on PD‐L1 expression. H) The interaction between PD‐L1 and SARs after the treatment of zosuqudiar was detected by Co‐IP assay. NCI‐H460 cells were transfected with PD‐L1‐HA plasmid for 24 h, followed by zosuqudiar and chloroquine (CQ) treatment. The cell lysates were immunoprecipitated with anti‐HA affinity beads. I) The colocalization of SQSTM1 and PD‐L1 in NCI‐H460 cells after the treatment of zosuquidar was detected by immunofluorescence. Scale bar: 10 µm. J) Depletion of *SQSTM1* influenced the effect of zosuquidar on PD‐L1 expression. NCI‐H460 cells were transfected with si*SQSTM1*, or siRNA‐negative control (NC) for 24 h as indicated, followed by treatment of zosuquidar. Data were presented as the mean ± SD. Unpaired 2‐tailed Student's *t*‐test (two groups) and one‐way ANOVA with Dunnett's post hoc test (more than two groups) were used to determine statistical significance of experimental data. ***, *P* < 0.001; **, *P* < 0.01; *, *P* < 0.05; ^###^, *P* < 0.001; n.s, not significantly different.

To explore the molecular mechanism of zosuquidar‐mediated PD‐L1 downregulation, we investigated the effect of zosuquidar on PD‐L1 protein stability and mRNA level. We found that zosuquidar significantly shortened the half‐life of PD‐L1 protein while had no effect on mRNA level. Moreover, the treatment of lysosome inhibitor NH_4_Cl, rather than proteasome inhibitor MG132, abolished zosuquidar‐mediated PD‐L1 degradation, suggesting that zosuquidar promoted the degradation of PD‐L1 through a lysosome‐dependent pathway (**Figure** **4**A and Figure S4A–D, Supporting Information).

Autophagy is a well‐conserved degradation process, proteins or entire organelles to be degraded are packaged by autophagosomes and transported to lysosomes for degradation.^[^ ^25^ ^]^ Thus, we asked whether the degradation of PD‐L1 mediated by zosuquidar is dependent on autophagy. To verify this hypothesis, LC3 was used as a marker of autophagy to investigate the effect of zosuquidar. As shown in Figure 4B,C, zosuquidar induced the expression of LC3‐II in a dose‐ and time‐dependent manner. Upregulated LC3‐II expression was also detected in 3 primary patient‐derived lung cancer cells (Figure S4E, Supporting Information). Considering that inhibition of autophagic flux can also induce the accumulation of LC3‐II, we transfected NCI‐H460 cells with LC3‐mCherry‐GFP plasmid to monitor autophagic flux, as mCherry and GFP fluorescent proteins showed different stability in acidic environment.^[^ ^26^ ^]^ The amount of autophagosome (yellow) and autolysosome (red) was increased after zosuquidar treatment, suggesting that zosuquidar could activate autophagy (Figure 4D). The transmission electron microscopy analysis also showed the activated autophagy in cells treated with zosuquidar (Figure 4E). Moreover, the colocalization between PD‐L1 and LC3 was enhanced by zosuquidar (Figure 4F). To further validate the role of autophagy in zosuquidar‐mediated PD‐L1 degradation, we respectively silenced *ATG5* (autophagy related 5) and *ATG7* (autophagy related 7) by siRNA, which are essential for the formation of autophagosome. As shown in Figure 4G, depletion of *ATG5* or *ATG7* disturbed zosuquidar‐induced PD‐L1 degradation. Together, these results demonstrated that zosuquidar reduced PD‐L1 expression through autophagy‐lysosome pathway.

Recent studies have revealed that autophagy can act as a highly selective degradation process by using a series of selective autophagy receptors (SARs), and the SQSTM1/p62 has been reported to be involved in sunitinib‐mediated PD‐L1 degradation.^[^ ^27^, ^28^ ^]^ To identify which SAR(s) might be involved in zosuquidar‐induced PD‐L1 autophagy degradation, we performed the co‐immunoprecipitation (Co‐IP) assay. Several classical SARs were included in our investigation, such as SQSTM1, TOLLIP (toll interacting protein), HDAC6 (histone deacetylase 6), and NBR1 (NBR1 autophagy cargo receptor). As shown in Figure 4H, only SQSTM1 showed an increased interaction with PD‐L1 in response to zosuquidar treatment. Induced colocalization of PD‐L1 and SQSTM1 after zosuquidar treatment was also detected by immunofluorescence assay (Figure 4I). Moreover, PD‐L1 degradation induced by zosuquidar was abolished by depletion of *SQSTM1*, while other SARs had no similar effect (Figure 4J and Figure S4F, Supporting Information). Taken together, these results indicated that zosuquidar promoted PD‐L1 degradation via SQSTM1‐dependent selective autophagy.

### 2.5 Zosuquidar Induced ER Retention of PD‐L1 Occurred before Autophagy Degradation

**Figure 5:**
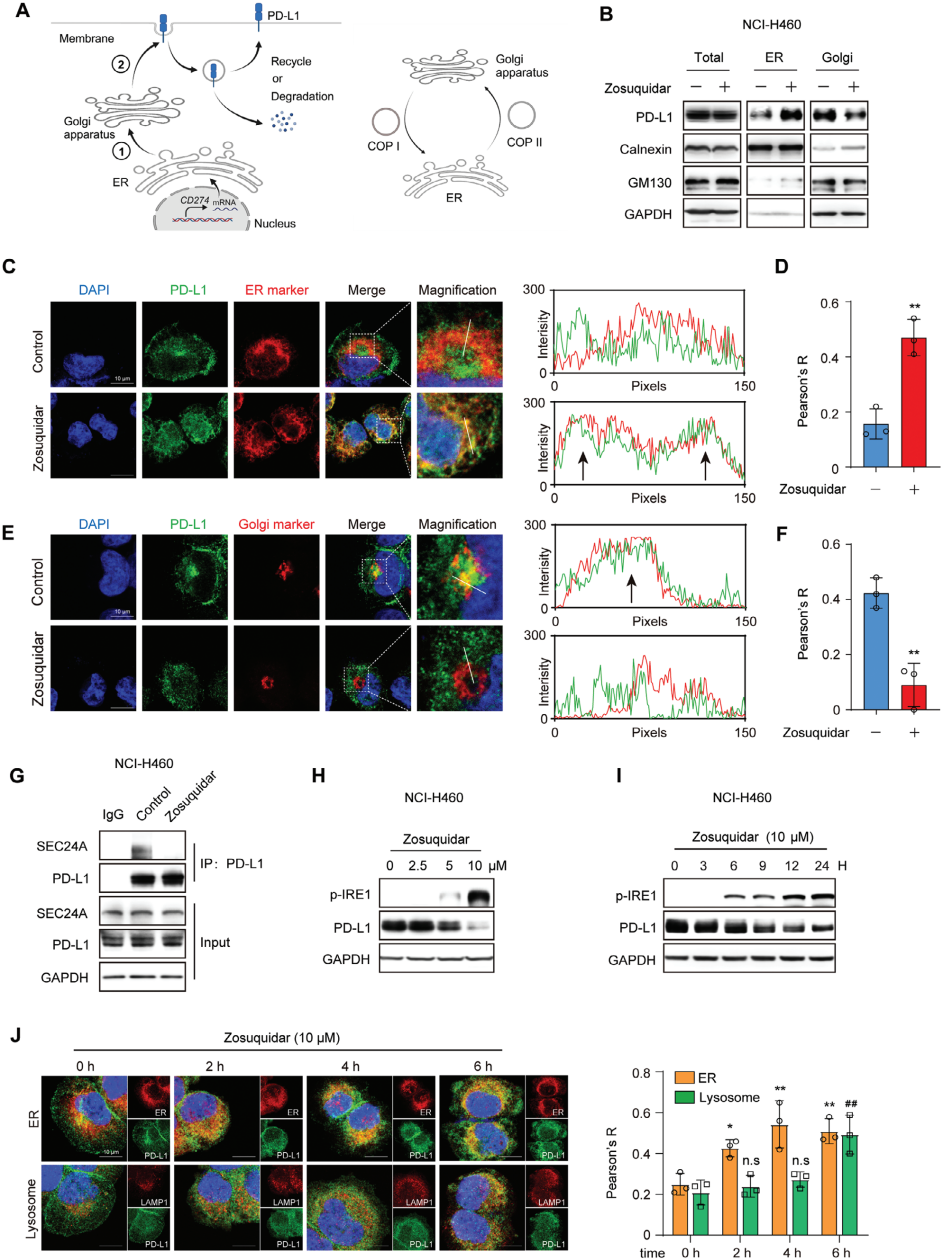
Zosuquidar disrupted PD‐L1 translocating from ER to Golgi apparatus. A) Schematic diagram of PD‐L1 synthesis, transportation, distribution, and degradation process (left), and diagram of COPI and COPII mediated protein ER‐Golgi transportation (right). B) The effect of zosuquidar on the distribution of PD‐L1 in ER and Golgi apparatus. NCI‐H460 cells were transfected with si*ATG5* RNA for 24 h, followed by the treatment of zosuquidar. The ER and Golgi apparatus protein fractions were separated according to the manufacturer's instructions. C–F) The colocalization of C) PD‐L1 and ER or E) Golgi apparatus in NCI‐H460 cells after the treatment of zosuquidar was detected by immunofluorescence. Scale bar: 10 µm. The intensity profiles of PD‐L1 and ER or Golgi apparatus along the white line were shown on the right. Blank arrowheads indicated colocalization. Pearson's *R* value of D) PD‐L1 and ER or F) PD‐L1 and Golgi apparatus were analyzed by Image J (*n* = 3). ER marker: Calnexin; Golgi apparatus marker: GM130. G) The interaction between PD‐L1 and SEC24A after the treatment of zosuqudiar was detected by Co‐IP assay. NCI‐H460 cells were treated with zosuqudiar and CQ (20 × 10^−6^ m) for 12 h and the cell lysates were immunoprecipitated with PD‐L1 antibody. H,I) The expression of p‐IRE1 in NCI‐H460 cells treated with different concentrations (2.5 × 10^−6^, 5 × 10^−6^, and 10 × 10^−6^ m) of H) zosuqudiar for 24 h or I) zosuqudiar (10 × 10^−6^ m) for 0, 3, 6, 9, 12, and 24 h. J) The colocalization of PD‐L1 and ER (Calnexin) or lysosome (Lamp1) in NCI‐H460 cells after the treatment of zosuquidar for 0, 2, 4, and 6 h. Scale bar: 10 µm. Pearson's *R* value was analyzed by Image J (*n* = 3). Data were presented as the mean ± SD. Unpaired two‐tailed Student's *t*‐test (two groups) and one‐way ANOVA with Dunnett's post hoc test (more than two groups) were used to determine statistical significance of experimental data. **, *P* < 0.01; *, *P* < 0.05; ^##^, *P* < 0.01; n.s, not significantly different.

Autophagy is an adaptive process that occurs in response to stress conditions such as nutrient deprivation, hypoxia, as well as protein aggregation.^[^ ^29^ ^]^ Therefore, we speculated whether zosuquidar promoted PD‐L1 autophagy degradation by triggering abnormal aggregation of PD‐L1. Considering that newly synthesized PD‐L1 will be sequentially transported to ER and Golgi apparatus for post‐translational modification before being located on the cell membrane (**Figure** **5**A), we separated ER protein fractions and Golgi protein fractions to evaluate the effect of zosuquidar on PD‐L1 intracellular distribution. Strikingly, zosuquidar treatment induced the accumulation of PD‐L1 in ER and reduced expression in Golgi apparatus (Figure 5B). Immunofluorescence assays also showed an increased localization of PD‐L1 to ER while the localization with Golgi apparatus decreased after the treatment of zosuquidar (Figure 5C–F). Meanwhile, the stability of PD‐L1, which has been transported to the plasma membrane, wasn't affected by the treatment of zosuquidar (Figure S5A,B, Supporting Information). Collectively, these findings suggest that zosuquidar caused ER retention of PD‐L1 before it is located on the cell surface.

PD‐L1 is extensively N‐glycosylated in the ER and Golgi apparatus. Aberrant glycosylated PD‐L1 will be trapped in ER and then degraded.^[^ ^30^ ^]^ Therefore, we speculated whether zosuquidar leaded the aberrant N‐glycosylation modification of PD‐L1, while the results showed unchanged glycosylation with the treatment of zosuquidar (Figure S5C, Supporting Information). Previous studies have identified that PD‐L1 trafficking from ER to Golgi apparatus is crucially dependent on the coat protein complex II (COPII) vesicles and the SEC24A, a core subunit of COPII, interacted with PD‐L1 thereby mediating its trafficking.^[^ ^21^ ^]^ Thus, we investigated whether ER retention of PD‐L1 induced by zosuquidar is associated with the disrupted interaction between PD‐L1 and SEC24A. Consistently, the treatment of zosuquidar severely suppressed the interaction between PD‐L1 and SEC24A (Figure 5G). Additionally, zosuquidar induced the expression of p‐IRE1 in a dose‐ and time‐dependent manner, suggesting the occurrence of ER stress with the treatment of zosuquidar (Figure 5H,I). Moreover, in order to explore the temporal relationship between PD‐L1 ER retention and autophagy degradation, we performed the confocal assay to investigate the colocalization of PD‐L1 to ER and lysosome (Lamp1) under different time treatments with zosuquidar. As shown in Figure 5J, increased colocalization between PD‐L1 and ER occurred 2 h after zosuquidar treatment, while the colocalization with lysosome appeared at 6 h. Taken together, these results indicated that the treatment of zosuquidar disrupted the interaction between PD‐L1 and SEC24A, leading to the retention of PD‐L1 in ER, which preceded PD‐L1 autophagy degradation.

### 2.6 Zosuquidar Promoted PD‐L1 Degradation through Its Pharmacological Target ABCB1

**Figure 6:**
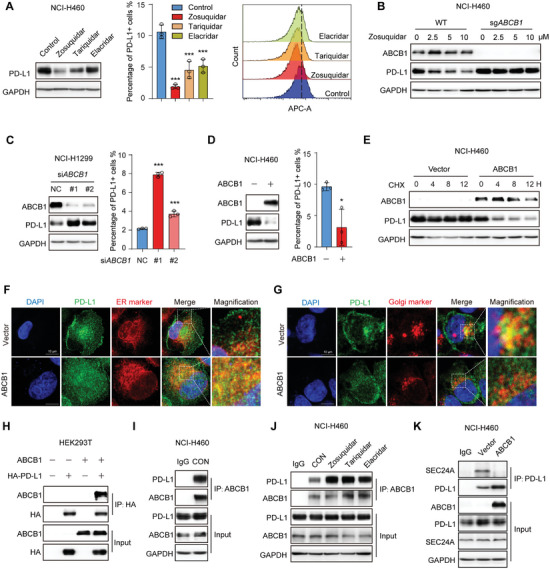
Zosuquidar promoted PD‐L1 degradation in an on‐target manner. A) Western blot (left) and flow cytometry analysis (right) of PD‐L1 expression in NCI‐H460 cells after the treatment of zosuquidar (10 × 10^−6^ m), tariquidar (10 × 10^−6^ m), and elacridar (10 × 10^−6^ m) for 24 h. B) Western blot of PD‐L1 expression in NCI‐H460 cells and crispr‐Cas9 generated *ABCB1* knockout NCI‐H460 cells after the treatment of zosuquidar. C,D) Western blot (left) and flow cytometry analysis (right) of PD‐L1 expression in NCI‐H1299 cells transfected with C) si*ABCB1* RNA or in NCI‐H460 cells after the transfection of D) ABCB1 plasmid for 24 h. E) Western blot of PD‐L1 protein half‐life in NCI‐H460 cells transfected with ABCB1 plasmid. The colocalization of F) PD‐L1 and ER or G) Golgi apparatus in NCI‐H460 cells transfected with ABCB1 plasmid was detected by immunofluorescence. ER marker: Calnexin; Golgi apparatus marker: GM130. Scale bar: 10 µm. H,I) The interaction between PD‐L1 and ABCB1 was detected by co‐IP assay. H) HEK293T was transfected with PD‐L1‐HA and ABCB1 plasmid as indicated and the cell lysate was immunoprecipitated with anti‐HA affinity beads. I) The NCI‐H460 cell lysate was immunoprecipitated with ABCB1 antibody. J) The effect of ABCB1 inhibitors on the interaction between PD‐L1 and ABCB1 was detected by Co‐IP assay. NCI‐H460 cells were treated with zosuquidar, tariquidar, and elacridar in the presence of CQ for 12 h, and the cell lysates were immunoprecipitated with ABCB1 antibody. K) The effect of ABCB1 on the interaction between PD‐L1 and SEC24A was detected by Co‐IP assay. NCI‐H460 cells were transfected with vector or ABCB1 plasmid in the presence of CQ for 12 h, and the cell lysates were immunoprecipitated with PD‐L1 antibody. Data were presented as the mean ± SD. Unpaired two‐tailed Student's *t*‐test (two groups) and one‐way ANOVA with Dunnett's post hoc test (more than two groups) were used to determine statistical significance of experimental data. ***, *P* < 0.001; *, *P* < 0.05.

Considering that zosuquidar is the selective inhibitor of ABCB1,^[^ ^31^ ^]^ we next investigated whether zosuquidar induced PD‐L1 degradation in an on‐target manner. We first applied another two ABCB1 inhibitors, tariquidar and elacridar, and found that both the total and surface expression of PD‐L1 were decreased with the treatment of ABCB1 inhibitors (**Figure** **6**A). Zosuquidar‐mediated PD‐L1 down‐regulation was also abolished when *ABCB1* was knocked‐out (Figure 6B). Furthermore, we silenced *ABCB1* by siRNA in NCI‐H1299 cell line, which has relatively high expression of ABCB1, and unexpectedly found that depletion of *ABCB1* significantly induced PD‐L1 expression (Figure 6C). In contrast, overexpression of ABCB1 decreased the expression of PD‐L1 and shortened its half‐life (Figure 6D,E). We further investigated the impact of ABCB1 on PD‐L1 intracellular distribution, and the immunofluorescence assays showed that ABCB1 overexpression significantly induced the ER retention of PD‐L1 and accompanied by decreased colocalization with Golgi apparatus (Figure 6F,G). Together, these results suggested that zosuquidar induced PD‐L1 degradation in a target‐dependent manner, and ABCB1 is a negative regulator of PD‐L1.

Next, we explored how ABCB1 negatively regulated PD‐L1 expression and why zosuquidar treatment and *ABCB1* depletion exhibited opposite effects on PD‐L1 expression. Given that ABCB1 is also a transmembrane protein, we speculated that ABCB1 may physically interact with PD‐L1. Consistently, the Co‐IP assay revealed that ABCB1 interacted with exogenous and endogenous PD‐L1 (Figure 6H,I). Thus, we asked whether the interaction between PD‐L1 and ABCB1 is involved in zosuquidar mediated PD‐L1 degradation. As shown by Co‐IP assay, the interaction between endogenous PD‐L1 and ABCB1 is significantly increased with the treatment of ABCB1 inhibitors, and the degree of increased interaction is consistent with their effect on PD‐L1 expression (Figure 6J). Moreover, the binding of PD‐L1 to SEC24A was impaired by ABCB1 overexpression (Figure 6K). Taken together, these results preliminary suggested that the interaction between PD‐L1 and ABCB1 may attenuate SEC24A‐mediated PD‐L1 transport, and the treatment of ABCB1 inhibitors enhanced PD‐L1‐ABCB1 interaction, leading to disruption of PD‐L1 transport from ER to Golgi apparatus and subsequent retention in the ER.

### 2.7 Zosuquidar Suppressed Tumor Growth via Its Target ABCB1

**Figure 7:**
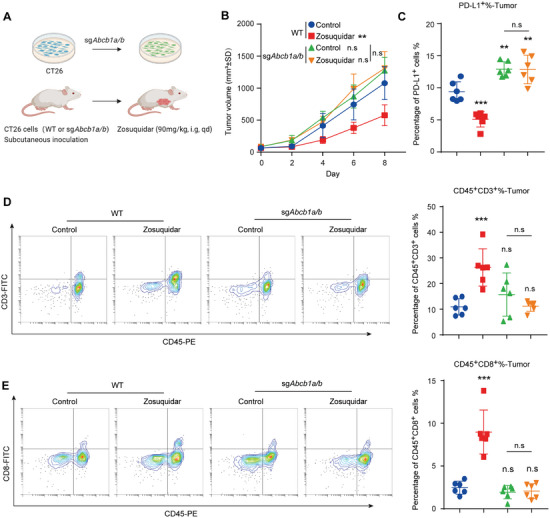
Zosuquidar suppressed tumor growth through ABCB1. A) Schematic illustration of crispr‐Cas9 generated *Abcb1a/b* knockout CT26 cells and CT26 mouse model. BALB/c mice were subcutaneously injected with CT26 cells (WT or sg*Abcb1a/b*) and treated with zosuquidar (90 mg kg^−1^, intragastric administration) daily for 8 d (*n* = 6). B) Tumor volume of each group was measured every 2 d with a caliper. C) Cell‐surface expression of PD‐L1 in each tumor tissues were determined by flow cytometry. D,E) Tumor infiltrating D) CD45^+^CD3^+^ T cells and E) CD45^+^CD8^+^ T cells were detected by flow cytometry. Representative flow cytometry results were shown on the left. Data were presented as the mean ± SD. One‐way ANOVA with Dunnett's post hoc test were used to determine statistical significance of experimental data. ***, *P* < 0.001; **, *P* < 0.01; n.s, not significantly different.

To further confirm that zosuquidar‐mediated PD‐L1 degradation is dependent on ABCB1, we constructed *Abcb1a/b* knockout CT26 cells by crispr‐Cas9 technique. Increased mPD‐L1 expression was detected in *Abcb1a/b* knockout CT26 cells, and the cell proliferation was not affected by the absence of ABCB1 in vitro (**Figure** **7**A, Figure S6A,B, Supporting Information). Subsequently, we subcutaneously inoculated wild‐type and *Abcb1a/b* knockout CT26 cells into BALB/c mice, and treated with or without zosuquidar respectively. As shown in Figure 7B, without zosuquidar treatment, the absence of ABCB1 lightly induced tumor growth, which may be associated with increased PD‐L1 expression when *Abcb1a/b* knocked out. Furthermore, the inhibitory effect of zosuquidar on tumor growth was eliminated in *Abcb1a/b* knockout group without affecting body weight (Figure 7B and Figure S6C, Supporting Information). Similarly, zosuquidar induced downregulation of PD‐L1 and increased infiltration of CD3^+^ and CD8^+^ T cells in tumor were also impaired when *Abcb1a/b* was knocked out (Figure 7C–E). Taken together, these results indicated that zosuquidar mediated PD‐L1 degradation and tumor growth inhibition were extremely dependent on the expression of ABCB1.

## 3 Discussion

**Figure 8:**
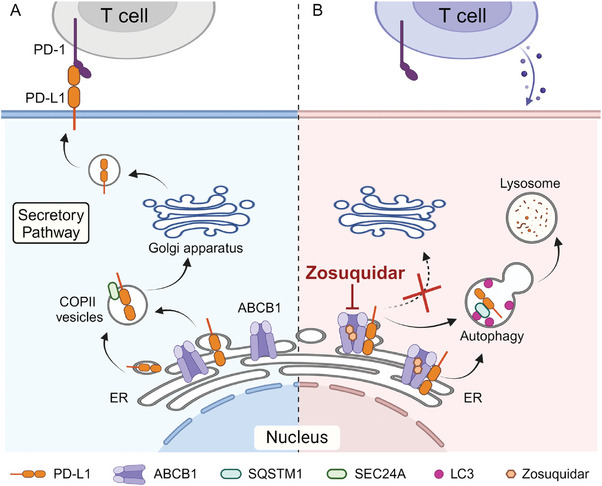
Schematic illustration for the mechanism of zosuquidar triggering PD‐L1 degradation. A) Newly synthesized PD‐L1 will be successively transported from ER to Golgi apparatus before it located at cell plasma membrane. Tumor cells with high expression of PD‐L1 on plasma membrane may evade T cell immune surveillance. B) The treatment of zosuquidar enhanced the interaction between ABCB1 and PD‐L1, which disrupted SEC24A mediated PD‐L1 transportation, and ultimately leaded selective autophagic degradation of PD‐L1. (Created with BioRender.com).

Blockade of PD‐1/PD‐L1 with monoclonal antibodies has broken the traditional pattern of cancer treatment and provided significant clinical benefits for cancer patients. However, increasing attention has been focused on the development of small molecule inhibitors targeting PD‐L1 expression as the intrinsic limitations of antibodies such as poor tissue permeability, frequent irAEs, and low response rate. Here, we found that zosuquidar, a ABCB1 inhibitor, significantly promoted PD‐L1 degradation, thus inducing the infiltration of cytotoxic T cell and repressing tumor growth in CT26 and humanized xenograft mouse model. Further mechanistic studies revealed that the interaction between ABCB1 and PD‐L1 was detrimental to the transport of PD‐L1 from ER to Golgi apparatus mediated by SEC24A, while zosuquidar induced the combination of ABCB1 and PD‐L1 thus triggered retention of PD‐L1 in ER and then degraded through SQSTM1‐dependent selective autophagy pathway (**Figure** **8**).

Zosuquidar is a highly selective modulator of ABCB1, that has been developed to overcome chemotherapy resistance mediated by ABCB1 expression.^[^ ^32^, ^33^ ^]^ By selectively occupying the cavity within ABCB1, zosuquidar significantly blocks ABCB1‐mediated drug efflux, thereby enhancing chemotherapy efficacy.^[^ ^34^, ^35^ ^]^ Zosuquidar has been studied in various clinical trials for its role in improving the sensitivity of chemotherapy. The combination of zoauquidar and chemotherapy such as daunorubicin and cytarabine were applied in patients with leukemia or acute myeloid leukemia (AML) to evaluate the efficacy of combination therapy compared to chemotherapy alone.^[^ ^36^, ^37^ ^]^ However, none or minor clinical benefits were obtained from these studies, and thus zosuquidar has not yet been approved for clinical use to date.^[^ ^38^, ^39^ ^]^ Studies have shown several reasons for the failure of ABCB1 inhibitors in clinical trials, such as without selecting patients with high levels of ABCB1 expression and the compensatory effects of other multidrug‐resistant proteins.^[^ ^39^ ^]^ Our study indicated zosuquidar could be an immunotherapy agent, which is against the fact that zosuquidar did not show significant benefit in clinical trials. The reason might be as following: 1) the highest concentration of zosuquidar we used in our study is 10 × 10^−6^ m, while the serum concentration in clinical is no more than 2 × 10^−6^ m;^[^ ^37^ ^]^ 2) our study indicates that zosuquidar exerts anti‐cancer effect through PD‐L1 degradation, which means only the patients with PD‐L1 expression could obtain benefits from zosuquidar. Therefore, the anti‐tumor activity of zosuquidar is extremely relies on the expression of PD‐L1 and the presence of immune system, which were not considered in previous clinical trials.^[^ ^40^ ^]^ Thus, the clinical benefits of zosuquidar might deserve further evaluation, and we provided a theoretical basis for its application in NSCLC therapy from an immune perspective.

ABCB1 is an ATP‐binding cassette (ABC) transporter that extrudes exogenous molecules by utilizing the energy from ATP hydrolysis.^[^ ^41^ ^]^ Many chemotherapy drugs have been identified as substrates of ABCB1. Therefore, high expression of ABCB1 typically leads to chemotherapy resistance,^[^ ^42^ ^]^ as it could extrude drugs to prevent them from reaching therapeutic concentrations in cancer cells. Up to now, the contribution of ABCB1 to multidrug resistance has been well evaluated, while its role in immune escape has not been determined. In this study, we identified ABCB1 as a negative regulator of PD‐L1, which interacted with PD‐L1 and hindered the transport of PD‐L1 from ER to Golgi apparatus. It remains unclear how ABCB1 interrupts the interaction between PD‐L1 and SEC24A in our work. We hypothesize that ABCB1 may occupy the binding region of PD‐L1 to SEC24A or generate steric hindrance due to its high molecular weight and multiple transmembrane structures. However, there is still a lack of direct evidence to support our hypothesis.

In addition, we also observed an enhanced binding between PD‐L1 and ABCB1 with the treatment of ABCB1 inhibitors, and the extent of increase is related to their suppressive effects on PD‐L1 expression. However, the mechanism of how inhibitors increase the interaction between PD‐L1 and ABCB1 remains unclear. As a transporter, ABCB1 extruded substrates with continuous conformational change.^[^ ^43^, ^44^ ^]^ Previous studies identified a distinct inward‐facing conformation of ABCB1 stabilized by zosuquidar as well as impaired homogeneous closing of the intracellular transmembrane domain and the formation of an outward‐facing conformation.^[^ ^45^ ^]^ Thus, we infer that the conformation of ABCB1 after inhibitors treatment has a higher affinity with PD‐L1, while we could not provide direct evidence to support this proposal. Of note, recent electron paramagnetic resonance (EPR) data showed that zosuquidar stabilized ABCB1 in a distinct higher energy state than tariquidar, and the cryo‐EM results showed a more noticeable rearrangement of the TM helices in zosuquidar‐bound ABCB1 than tariquidar.^[^ ^34^, ^46^ ^]^ Whether the conformation change of ABCB1 mediated by inhibitors is associated with our findings deserves further investigation.

Accumulated evidence has highlighted the crucial role of selective autophagy in regulating PD‐L1 expression and PD‐L1‐dependent immune escape, while the relevant mechanisms have not been clarified. Up to now, several membrane compartments including Golgi apparatus,^[^ ^47^ ^]^ mitochondria,^[^ ^18^ ^]^ and endosomes.^[^ ^17^ ^]^ have been identified to be involved in the autophagic degradation of PD‐L1, and the SQSTM1.^[^ ^27^ ^]^ is characterized as the autophagy cargo receptor mediating PD‐L1 autophagy degradation. By triggering PD‐L1 autophagy degradation, both sunitinib and amlodipine promoted anti‐tumor immunity. Here, we showed that zosuqudiar caused the abnormal aggregation of PD‐L1 in ER, which was subsequently degraded by the SQSTM1‐dependent selective autophagy. The treatment of zosuquidar also significantly suppressed tumor growth in vivo.

Recent studies have suggested that exhausting the expression of PD‐L1 throughout the whole cell seems to be a more attractive way compared to blocking the biological function of PD‐L1 on the cell surface, as the redistribution and compensatory synthesis of PD‐L1 may contribute to acquired resistance against anti‐PD‐L1 mAbs. Here we showed that zosuquidar triggered PD‐L1 degradation in the early secretory pathway, which would contribute to deplete intracellular PD‐L1 before it is transported to the cell membrane. The in vivo experiments also showed that zosuquidar achieved significant tumor suppression effects. Collectively, our study suggested that intervening the maturation and subcellular transportation of PD‐L1 is a promising immunotherapy strategy and provided evidence for the potential application of zosuquidar in immunotherapy.

In summary, we identified zosuquidar as an effective PD‐L1 degrader, which disrupted the transport of PD‐L1 from ER to Golgi apparatus and promoted PD‐L1 degradation through SQSTM1‐dependent selective autophagy pathway. We also revealed the important function of ABCB1 in negatively regulating PD‐L1 expression. Taken together, our work provides an effective strategy to triggering PD‐L1 degradation from the perspective of intervening in PD‐L1 transportation.

## 4 Experimental Section

### Antibodies for Immunoprecipitation and Immunoblotting

Rabbit anti‐PD‐L1 (13684), rabbit anti‐LC3B (2775s), rabbit anti‐HDAC6 (7558s), and rabbit anti‐ABCB1 (13342s) were purchased from Cell Signaling Technology (Boston, USA). Rabbit anti‐GAPDH (db106), and rabbit anti‐HA (db2603) were from Diagbio. Rabbit anti‐ATG5 (10181‐2‐AP), rabbit anti‐ATG7 (10088‐2‐AP), rabbit anti‐SQSTM1 (18420‐1‐AP), rabbit anti‐Calnexin (10427‐2‐AP), rabbit anti‐NBR1 (16004‐1‐AP), rabbit anti‐TOLLIP (11315‐1‐AP), and rabbit anti‐SEC24A (15958‐1‐AP) were obtained from Proteintech (Chicago, USA). Mouse anti‐GM130(ab169276), and Rabbit anti‐IRE1 (phospho S724) (ab243665) were from Abcam (Cambridge, UK).

### Antibodies for Immunofluorescence

Rabbit anti‐PD‐L1 (ab213524), and mouse anti‐GM130(ab169276) were from Abcam (Cambridge, UK). Rabbit anti‐SQSTM1 (18420‐1‐AP), and rabbit anti‐Calnexin (10427‐2‐AP) were from Proteintech (Chicago, USA). Mouse anti‐PD‐L1 (TA809809) was from OriGene (Maryland, USA). Mouse anti‐LC3B (sc‐376404) was from Santa Cruz Biotechnology (TEXAS, USA). Mouse anti‐Lamp1 (14‐1079‐80) was purchased from eBioscience (California, USA).

### Antibodies for Flow Cytometry

FITC mouse IgG1 κ isotype control (400110), PerCP‐Cy5.5 mouse IgG1 κ isotype control (400149), PE mouse IgG1 κ isotype control (400114), PerCP‐Cy5.5 anti‐human CD3 (300430), FITC anti‐human CD8a (300906), PE anti‐human CD45 (368510), FITC Rat IgG2a κ isotype control (400506), FITC Rat IgG2b, κ isotype control (400605), PE Rat IgG2b, κ isotype control (400607), FITC anti‐mouse CD3 (100204), FITC anti‐mouse CD8a (100705), PE anti‐mouse CD45 (103106), and PE anti‐mouse PD‐L1 (124307) were purchased from Biolegend (Chicago, USA). APC anti‐human PD‐L1 (563741), PE anti‐human PD‐L1 (557924), PE Mouse IgG1, κ isotype control (559320), and APC Mouse IgG1, κ isotype control (554681) were from BD Biosciences (State of New Jersey, USA).

### Reagents

The library of anti‐cancer drugs which contains 320 chemical compounds dissolved at 10 × 10^−3^ m in dimethyl sulfoxide (DMSO) was purchased from TargetMol (Shanghai, China). Amlodipine (T1385), zosuqudiar trihydrochloride (T6018), chloroquine (T8689), and MG132 (T2154) were purchased from TargetMol (Shanghai, China). NH_4_Cl (326372), sulforhodamine B (230162), and cycloheximide (239763‐M) were purchased from Sigma‐Aldrich (Saint Louis, USA). IFN‐γ was purchased from PeproTech China (300‐02‐1000 ug). DAPI (C1002), and 4% paraformaldehyde fix solution (P0099) were purchased from Beyotime (Zhengzhou, China).

### Cell Culture

All the cell lines were kindly provided by Cell Bank of Shanghai Institutes for Biological Sciences, Chinese Academy of Sciences (Shanghai, China). All cell lines were tested and verified to be free of Mycoplasma. NCI‐H1299, NCI‐H460, NCI‐H1975, NCI‐H292, HCC827, SKOV3, 786‐O, and CT26 were cultured in RPMI‐1640 (11875093, Gibco) supplemented with 10% fetal bovine serum (FBS; Hyclone, SV30160.03). MIA PaCa‐2 and HEK293T were maintained in DMEM (Gibco, 11965092) with 10% FBS. All the cell lines were cultured in a humidified incubator at 37 °C in 5% CO_2_.

### Primary Cancer Cell Isolation

The fresh tumor tissues were obtained from NSCLC patients which has been approved by Zhejiang Cancer Hospital Committee (IRB‐2019‐175). The cancer tissues were cleaned twice with RPMI‐1640 to remove the non‐tumor tissues and blood stasis on the surface, and then physically dissociated into rice‐grain‐sized pieces with sterile surgical instruments. The tumor tissue blocks were subsequently cultured in DMEM/F‐12 (Gibco, 11320033) medium with 20% FBS and the medium was changed every 2 d until isolated cancer cells grew out of the tissue. Cells were maintained in a humidified incubator at 37 °C in 5% CO_2_.

### Western Blot

Cells were washed twice with cold PBS and lysed 1% NP‐40 buffer (25 mmol L^−1^ Tris‐base, pH 7.4, 150 mmol L^−1^ NaCl, 10% glycerol). Equal quantities of cell lysates were loaded into SDS‐PAGE gels for electrophoresis. After electrophoresis, proteins were transferred to 0.45 µm PVDF membranes and incubated with indicated primary antibodies and HRP‐conjugated secondary antibody successively. Immunoblotting images were acquired using the chemiluminescence imaging system (GE Amersham Imager 600, USA).

### Flow Cytometry

Cells were separated into single‐cell suspension and then labeled with APC‐conjugated PD‐L1 at 4 °C for 2 h (5 µL / 2 × 10^5^ cells). Samples were washed with PBS and then detected by CytoFLEX flow cytometry (Bechman, USA). Tumor tissues were minced and separated into single‐cell suspension through a tissue‐dissociator (Miltenyi Biotec, USA), and then stained with antibodies against CD3, CD8a, and CD45 for 30 min at room temperature (5 µL / 2 × 10^5^ cells) or antibody against PD‐L1 for 2 h at 4 °C. Samples were washed with PBS and analyzed by flow cytometry.

### Protein Half‐Life Assay

Cells were seeded on six‐well plates for 24 h and then treated with DMSO, MG132, or NH_4_Cl. Subsequently, cells were treated with cycloheximide (CHX, 10 µg mL^−1^) or CHX + zosuquidar (10 × 10^−6^ m) for different time points as indicated in the figure legends. Alternatively, cells were transfected with empty vector or ABCB1 plasmid for 24 h and treated with CHX for different time points. Cells were collected and determined by western blot assay as mentioned above. Image J was used for grayscale analysis.

### RNA Isolation and Quantitative Real‐Time PCR

Cells were seeded on six‐well plates and treated with different concentrations of zosuquidar for 24 h. Total RNA was extracted using TRIzol reagent (TaKaRa, 9109) and purified according to the standard protocol. NanoDropND‐1000 spectrophotometer (Thermo Fisher Scientific, USA) was used to detect RNA concentration, and 2 µg purified total RNA was used to synthesize cDNA by using TransScript One‐Step gDNA Removal and cDNA Synthesis SuperMix (TRAN, AT311‐03) according to the manufacturer's instructions. Quantitative PCR was performed with SYBR‐Green kit (Bio‐Rad, 172‐5124), and the comparative Ct method was used to calculate the quantification of the target genes. The quantitative results were presented as fold change which were normalized to *ACTB* mRNA levels. Primers pairs used for quantitative real‐time PCR are as follows: human *CD274*: forward, 5′‐TCACTACACAGCCCTCCTAA‐3′, reverse, 5′‐ACACCAGAATATGGCCAAGAG‐3′; human *ACTB*: forward, 5′‐ATTCCTATGTGGGCGACGAG‐3′, reverse, 5′‐CCAGATTTTCTCCATGTCGTCC‐3′.

### High Content Screening.^[^ ^48^ ^]^

NCI‐H1299 cells were seeded in 96‐well plates and transfected with PD‐L1‐GFP for 24 h. After transfection, cells were treated with the anti‐cancer drugs library containing 320 chemical compounds (10 × 10^−6^ m) for 24 h, followed by fixed with 4% paraformaldehyde for 20 min at room temperature. Nucleus were stained with DAPI (1:1000). Images were captured by ImageXpress Pico system (Molecular Devices, China) at 10× magnification, and analyzed by using CellReporterXpress software. The fluorescence intensity was normalized to control group and presented as fold change. The results of 19 compounds were not presented due to severe cytotoxicity.

### siRNA‐Mediated Silencing

Cells were plated in six‐well plates for 24 h and then transfected with transfection reagent JetPRIME (Polyplus, 409‐10) and target siRNA (*ATG5*, *ATG7*, *SQSTM1*, *HDAC6*, *NBR1*, *TOLLIP*, and *ABCB1*) or siRNA‐negative control (NC) according to the manufacturer's instructions for 24 h. The siRNA sequences used are listed in Table S2 (Supporting Information).

### Plasmid Construction

Cells were plated on six‐well plates or 100 mm × 100 mm cell culture dishes for 24 h and subsequently transfected with indicated plasmids or empty vector by using transfection reagent JetPRIME (Polyplus, 114‐15) according to the manufacturer's instructions for 24 h. The PD‐L1‐GFP (EX‐U0767‐M98) and ABCB1 (EX‐E2266‐M02‐B) were synthesized by Guangzhou FulenGen (Guangzhou, China). LC3B‐GFP‐mCherry (P0446) was synthesized by MiaoLingPlasmid (Wuhan, China). The PD‐L1‐HA was generated by inserting PCR‐amplified *CD274* cDNA, which was synthesized by using KOD‐Plus‐Neo kit (Toyobo, F1066K) into the pcDNA3.0‐HA vector.

### Immunofluorescence Assay

Cells were seeded in eight‐well Lab‐Tek II chamber slide (Thermo Fisher Scientific, 154534) for 24 h, and then treated with zosuquidar or transfected with plasmids as indicated in the figure legends. Cells were fixed with 4% paraformaldehyde for 20 min at room temperature, followed by permeabilized with 0.1% Triton X‐100 in PBS at 4 °C for 10 min. After being blocked by 4% bovine serum albumin (BSA) for 30 min at 37 °C, the cells were incubated with indicated primary antibodies at 4 °C overnight, and subsequently incubated with Alexa Fluor 488‐ or Alexa Fluor 568‐conjugated secondary antibodies (Thermo Fisher Scientific, A11001, A11004, A11011, A11008; 1:200) for 1 h at room temperature. Nucleus were stained with DAPI (1:1000) for 15 min at room temperature. Images were captured by confocal microscopy (Leica TCS SP8, Germany). The plugin “coloc2” of Image J was used to calculate the colocalization factor (Pearson's R value) and the intensity profiles were analyzed by “plot profile” module in Image J.

### Immunoprecipitation Analyses

For exogenous coimmunoprecipitation experiments, HEK293T cells were seeded in 100 mm × 100 mm dishes for 24 h, followed by transfected with indicated plasmids or empty vector. Cells were lysed by lysis buffer (25 mmol L^−1^ Tris‐base, 150 mmol L^−1^ NaCl, 1% NP‐40, 1 mmol L^−1^ PMSF, 1 mmol L^−1^ Na_3_VO_4_, and 5 µg mL^−1^ leupeptin, pH 7.4), and the protein concentration was measured by BCA protein quantification kit (Yeasen Biotech, China) according to the manufacturer's instructions. Lysates were incubated with anti‐HA affinity beads at 4 °C overnight, followed by immunoblotting as described.

For endogenous coimmunoprecipitation experiments, H460 cells were seeded in 100 mm × 100 mm dishes for 24 h, followed by treated with ABCB1 inhibitors as indicated in figure legends. Cells were lysed by lysis buffer (25 mmol L^−1^ Tris‐base, 150 mmol L^−1^ NaCl, 1% NP‐40, 1 mmol L^−1^ PMSF, 1 mmol L^−1^ Na_3_VO_4_, and 5 µg mL^−1^ leupeptin, pH 7.4), and the protein concentration was measured by BCA protein quantification kit according to the manufacturer's instructions. Cell lysates were incubated with indicated antibodies at 4 °C overnight, and then pulled down with protein A/G beads (Santa Cruz Biotechnology, sc‐2003) at 4 °C for 2 h.

### CRISPR/Cas9‐Mediated KO of ABCB1

PX458‐*ABCB1* sgRNAs (PX458‐*Abcb1a* sgRNAs or PX458‐*Abcb1b* sgRNAs) were generated by inserting targeted sgRNAS into Bbs1‐digested PX458 plasmid. Cells were electroporated with indicated PX458‐sgRNA for 24 h and detected by flow cytometry. The fluorescence‐positive cells were considered to have successfully transfected with the indicated plasmid. Single cells were sorted and plated in individual wells of the 96‐well plates, and the clones were obtained for few days culture. The sgRNA oligonucleotide sequences are as follows:

human *ABCB1* sgRNA:
forward, 5′‐CACCGCAACAACCCTGTGGTCCATC‐3′,
reverse, 5′‐ AAACGATGGACCACAGGGTTGTTGC‐3′;
mouse *Abcb1a* sgRNA:
forward, 5′‐CACCGAGCATATGATGCATAGATA‐3′,
reverse, 5′‐AAACTATCTATGCATCATATGCTC‐3′;
mouse *Abcb1b* sgRNA:
forward, 5′‐ CACCGCATATGATGCATAGACCAAC‐3′,
reverse, 5′‐AAACGTTGGTCTATGCATCATATGC‐3′.

### Transmission Electron Microscopic Analysis

Cells were fixed in 2.5% glutaraldehyde solution (Sigma‐Aldrich, G5882) for 24 h at 4 °C, and then fixed with 1% osmic acid for 1 h at room temperature. Samples were subsequently treated with 2% uranyl acetate for 30 min at room temperature, followed by dehydration and ultrathin section (Leica UC7). The ultrathin sections were stained with lead citrate and uranyl acetate, and observed with Tecnai spirit 120kv electron microscope (Thermo Scientific, USA).

### ER and Golgi Apparatus Fractions Isolation

Cells were seeded in 100 mm × 100 mm cell culture dishes and transfected with siATG5 followed by treatment of zosuquidar for 12 h. The ER and Golgi apparatus fractions were isolated by using Minute ER Enrichment Kit (Invent, ER‐036) and Minute Golgi apparatus Enrichment Kit (Invent, GO‐037) according to the manufacturer's instructions.

### Cell Proliferation Assay

Cells were seeded in 96‐well plates at a density of 600 cells per well, and fixed with 10% (w/v) trichloroacetic acid at indicated times. After washed with running tap water, cells were stained with sulforhodamine B for 30 minutes at room temperature. The excess dye was washed with 1% (v/v) acetic acid and then the protein‐bound dye was dissolved by 10 × 10^−3^ m Tris‐base. OD values were measured at 540 nm by microplate reader (Molecular Devices).

### Membrane Protein Degradation Assay

Cells were separated into single‐cell suspension and then labeled with APC‐conjugated PD‐L1 at 4 °C for 2 h (5 µL / 2 × 10^5^ cells in 100 µL 0.2% BSA). After washed with PBS, cells were cultured in RPMI‐1640 medium with 10% FBS at 37 °C for indicated time in the presence or absence of zosuquidar (10 × 10^−6^ m). Samples were washed with PBS and then detected by CytoFLEX flow cytometry (Bechman, USA).

### Animal Experiments

To establish CT26 mouse model, CT26 cells (5 × 10^5^ cells per mouse in 100 µL RPMI‐1640 medium) were collected and injected subcutaneously in 6 week old BALB/c female mice. Tumor volumes were measured every 2 d with a caliper and calculated by the formula: length × width^2^ × 0.5. Mice were randomly divided into 3 groups when tumor volume reached 50–100 mm^3^ and grouped into control, zosuquidar, and anti‐mouse PD‐1 treatment. Vehicle or zosuquidar (90 mg kg^−1^) was orally treated daily, and anti‐mouse PD‐1 treatment was given twice a week by intravenous injection (5 mg kg^−1^). Mice were euthanized if the tumor volume exceeded 1500 mm^3^ or the occurrence of ulceration.

4 week old female NSG (NOD‐*Prkdc^scid^Il2rg^em1^/Smoc*) mice were purchased from Model Organism Center Inc for establishing the PBMC‐based humanized xenograft model. H292 cells (5 × 10^6^ cells per mouse in 100 µL RPMI‐1640 medium) were injected subcutaneously into NSG mice. Mice were tail vein injected with human PBMCs (peripheral blood mononuclear cells, 1.5 × 10^7^ per mouse) when tumors were formed. The humanization states of each mouse were qualified by using flow cytometry to detect the percentage of hu‐CD45^+^ cells in mouse peripheral blood. Mice were randomly divided into three groups when tumor volume reached roughly 100 mm^3^ and grouped into control, zosuquidar, and anti‐human PD‐1 treatment. Vehicle or zosuquidar (90 mg kg^−1^) was orally treated daily, and anti‐human PD‐1 treatment was given twice a week by intravenous injection (5 mg kg^−1^). Mice were euthanized 12 d after drug treatment or if the tumor volume exceeded 1500 mm^3^.

### Statistical Analysis

All the statistical data were presented as the mean ± SD of triplicate independent experiments. Statistical analyses were performed by GraphPad Prism v.8.0 and *P* < 0.05 was considered statistically significant. Unpaired two‐tailed Student's *t*‐test (two groups), one‐way ANOVA with Dunnett's post hoc test (more than two groups) and log rank test were used to determine statistical significance of experimental data.

### Ethical Approval

In order to evaluate the effect of zosuquidar on tumor growth, CT26 mouse model and PBMC‐based humanized xenograft model were performed. The animal studies were performed according to the guidelines approved by the Institutional Animal Care and Use Committee of Zhejiang University (IACUC: 23‐197).

## Conflict of Interest

The authors declare no conflict of interest.

## Author Contributions

L.D. and H.G. contributed equally to this work. L.D. and H.G. designed the research and wrote the manuscript; H.G. performed experiments of the work; J.Z., M.Z., and W.Z. assisted with Western blotting and cell culture; L.W. and Q.D. assisted with in vivo experiments; C.Z. and H.W. contributed to statistical analysis and revised the manuscript; Y. X. contributed to the collection of patient samples. Q.H. and B.Y. conceptualized the work and provided project supervision.

## Supporting information

Supporting Information

## Data Availability

The data that support the findings of this study are available from the corresponding author upon reasonable request.
