# Supplementary material for: Zosuquidar Promotes Antitumor Immunity by Inducing Autophagic Degradation of PD‐L1
Source: Adv Sci (Weinh). 2024 Sep 4;11(41):2400340. doi: 10.1002/advs.202400340 (PMC11538701; doi:10.1002/advs.202400340)
Supplement: Supplementary file 1 — Supporting Information [file ADVS-11-2400340-s001.docx]

Supporting Information

**Zosuquidar Promotes Anti-tumor Immunity by Inducing** **Autophagic Degradation of PD-L1**

*Ling Ding ^#^, Hongjie Guo ^#^, Jie Zhang, Mingming Zheng, Wenjie Zhang, Longsheng Wang, Qianqian Du, Chen Zhou, Yanjun Xu, Honghai Wu, Qiaojun He*, and Bo Yang**


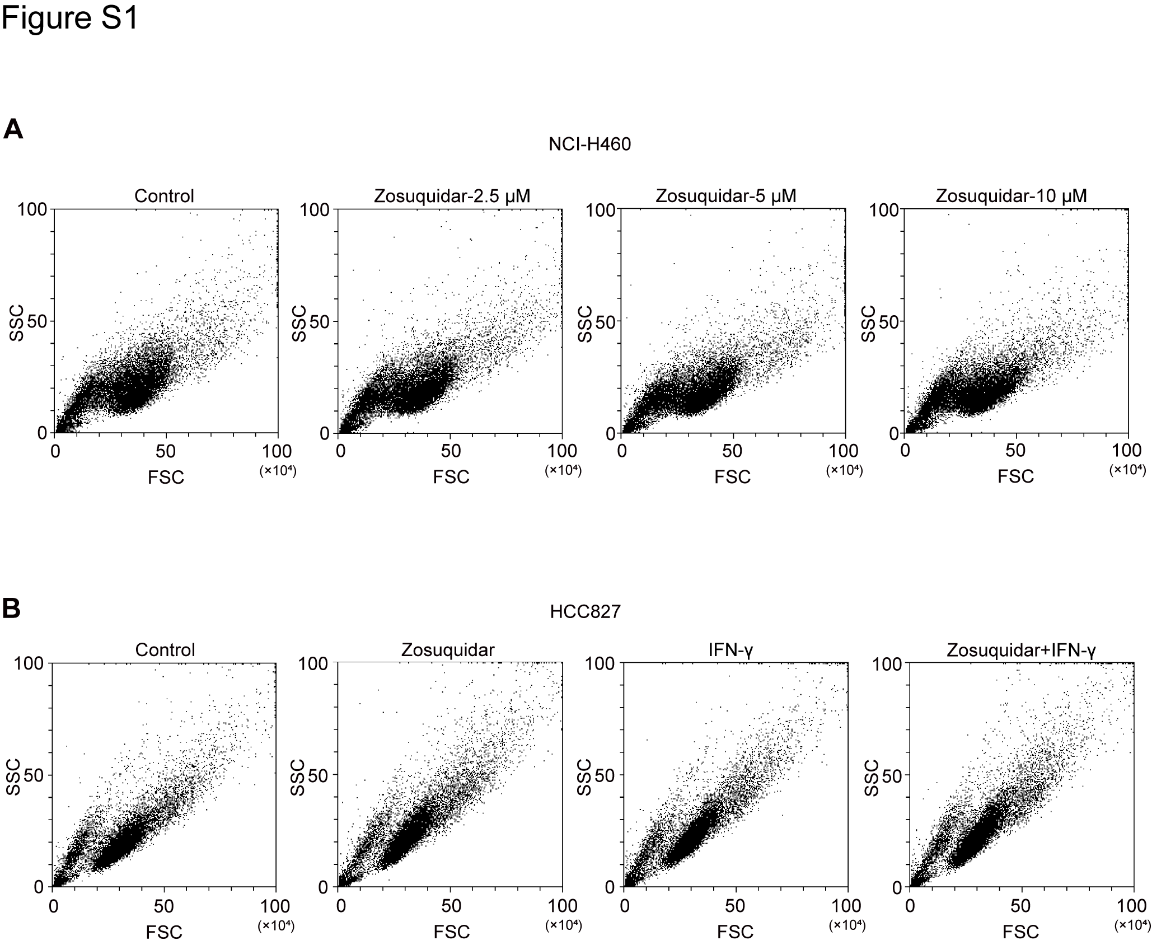


**Figure S1.** Representative original dot plots for flow cytometry assay. (A) NCI-H460 cells were treated with different concentrations (2.5 μM, 5 μM, and 10 μM) of zosuqudiar for 24 hours. (B) HCC827 cells were treated with zosuquidar (10 μM) alone or together with IFN-γ (10 ng/mL) for 24 hours.


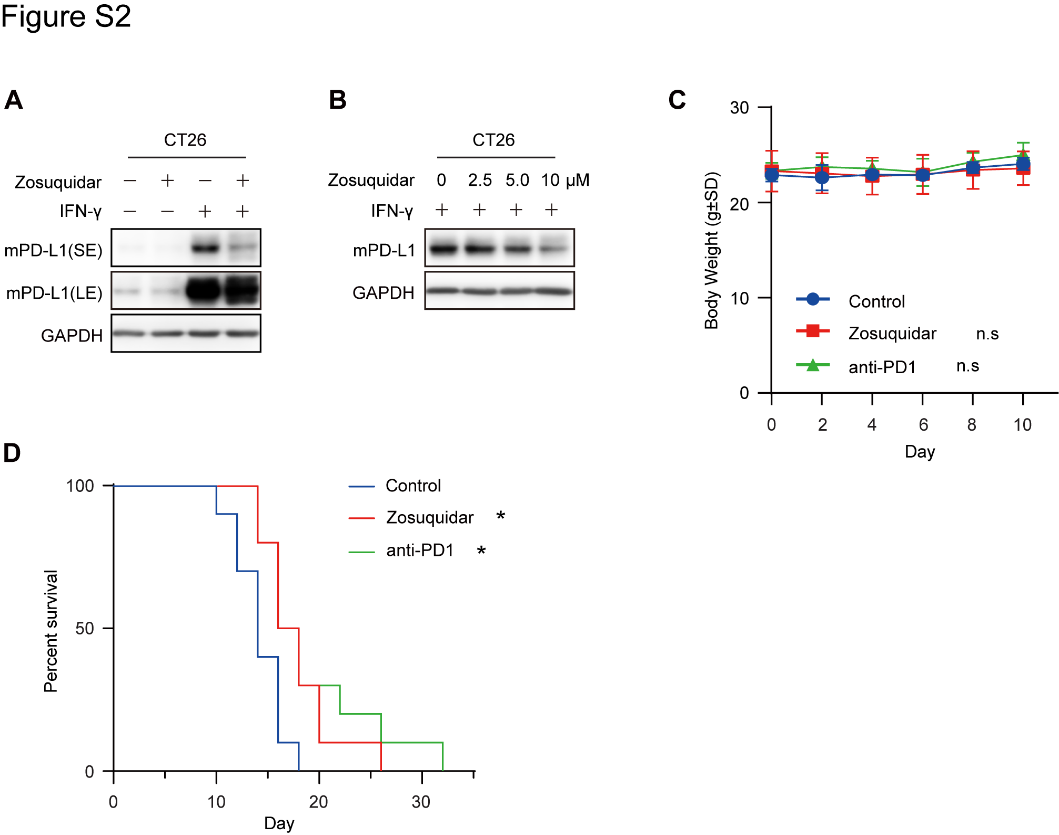


**Figure S2.** The treatment of zosuquidar increased animal survival in CT26 mouse model. (A) Western blot of mPD-L1 expression in CT26 cells treated with zosuquidar (10 μM) alone or together with IFN-γ (10 ng/mL) for 24 hours. LE: long exposure; SE: short exposure. (B) Western blot of mPD-L1 expression in CT26 cells treated with different concentrations (2.5 μM, 5 μM, and 10 μM) of zosuqudiar for 24 hours in the presence of IFN-γ. (C) Body weight of each group in CT26 mouse model was measured every two days. (D) Survival analysis conducted on CT26 mouse model. BALB/c mice were subcutaneously injected with CT26 cells and treated with zosuquidar (90 mg/kg, intragastric administration) daily or anti-PD-1 (5 mg/kg, intravenous injection) twice a week (*n* = 10). Data were presented as the mean ± SD. One-way ANOVA with Dunnett’s post hoc test and log rank test were used to determine statistical significance of experimental data. *, *P* < 0.05; n.s, not significantly different.


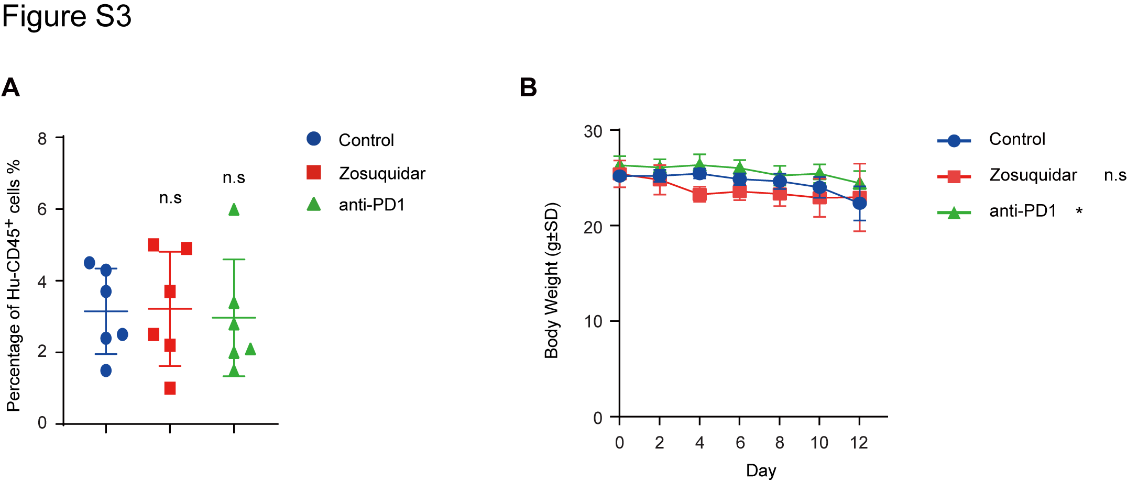


**Figure S3.** PBMC-based humanized xenograft model was used to evaluate the anti-tumor efficacy of Zosuquidar. (A) Hu-CD45^+^ T cells in the peripheral blood were detected by flow cytometry. (B) Body weight of each group was measured every two days. Data were presented as the mean ± SD. One-way ANOVA with Dunnett’s post hoc test was used to determine statistical significance of experimental data. *, *P* < 0.05; n.s, not significantly different.


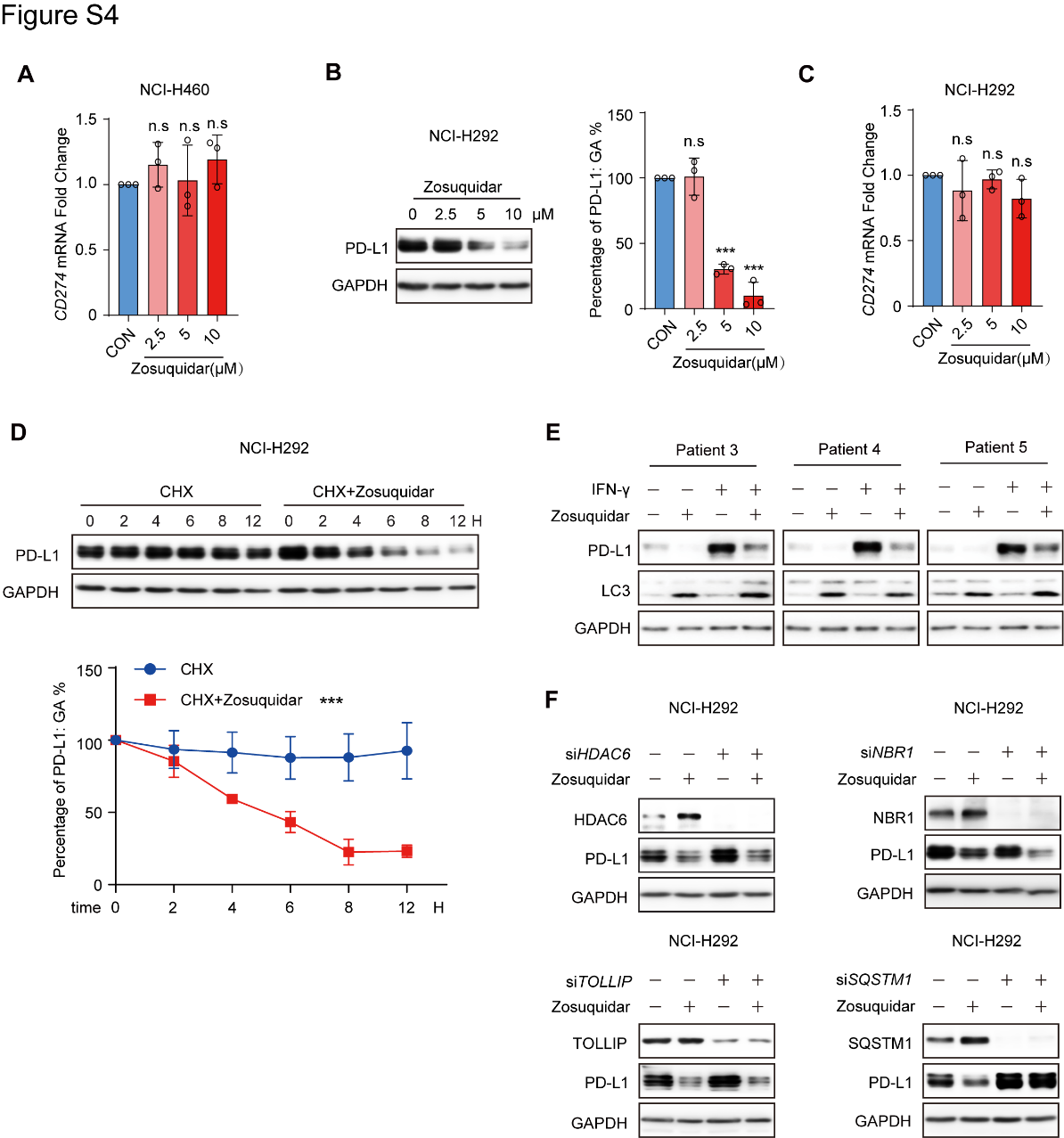

**Figure S4.** Zosuquidar triggered PD-L1 degradation through SQSTM1-dependent autophagy. (A) qRT-PCR analysis of *CD274* mRNA levels in NCI-H460 cells treated with different concentrations (2.5 μM, 5 μM, and 10 μM) of zosuqudiar for 24 hours. (B) Representative western blot (left) of PD-L1 expression in NCI-H292 cells treated with indicated concentrations of zosuqudiar for 24 hours, and quantified using Image J grayscale analysis (right). (C) qRT-PCR analysis of *CD274* mRNA levels in NCI-H292 cells treated with indicated concentrations of zosuqudiar for 24 hours. (D) Representative western blot of PD-L1 protein half-life in NCI-H292 cells treated with cycloheximide (10 μg/mL) alone or cycloheximide (10 μg/mL) plus zosuquidar (10 μM) for indicated time (top), and quantified using Image J grayscale analysis (bottom). (E) Western blot of PD-L1 and LC3 expression in 3 human primary patient-derived lung cancer cells with the treatment of zosuquidar (10 μM) alone or together with IFN-γ (10 ng/mL) for 24 hours. (F) Western blot of PD-L1 expression in NCI-H292 cells treated with zosuquidar alone or in the presence of indicated SARs depletion. NCI-H292 cells were transfected with si*HDAC6*, si*NBR1*, si*TOLLIP*, si*SQSTM1*, or siRNA-negative control (NC) for 24 hours as indicated, followed by treatment of zosuquidar. Data were presented as the mean ± SD. Unpaired 2-tailed Student’s *t*-test (two groups) and One-way ANOVA with Dunnett’s post hoc test (more than two groups) were used to determine statistical significance of experimental data. ***, *P* < 0.001; n.s, not significantly different.


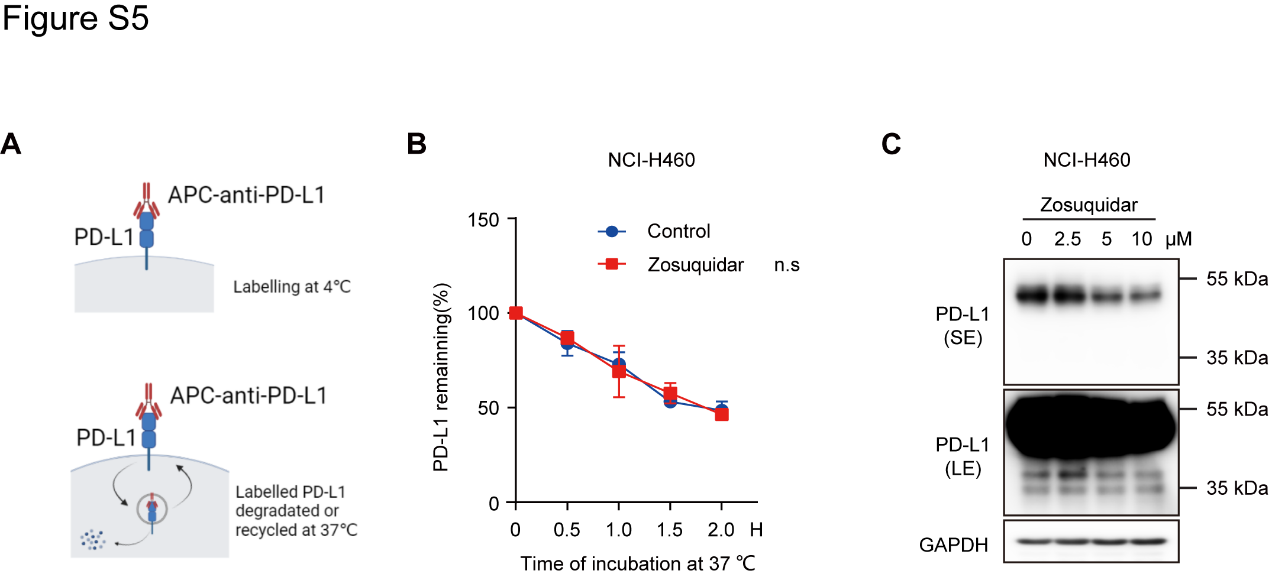


**Figure S5.** Zosuquidar does not affect PD-L1 stability on the plasma membrane and N-glycosylation modification. (A) Schematic illustration of membrane protein degradation assay detected by flow cytometry. (B) Flow cytometry analysis of PD-L1 stability on the cell surface in NCI-H460 cells treated with zosuquidar for indicated time. NCI-H460 cells labeled with APC-conjugated PD-L1 were cultured in RPMI-1640 medium with 10% FBS at 37 °C for indicated time in the presence or absence of zosuquidar (10 μM). (C) Western blot PD-L1 expression in NCI-H460 cells treated with different concentrations (2.5 μM, 5 μM, and 10 μM) of zosuqudiar for 24 hours; SE: short exposure; LE: long exposure. Data were presented as the mean ± SD. Unpaired 2-tailed Student’s *t*-test was used to determine statistical significance of experimental data. n.s, not significantly different.


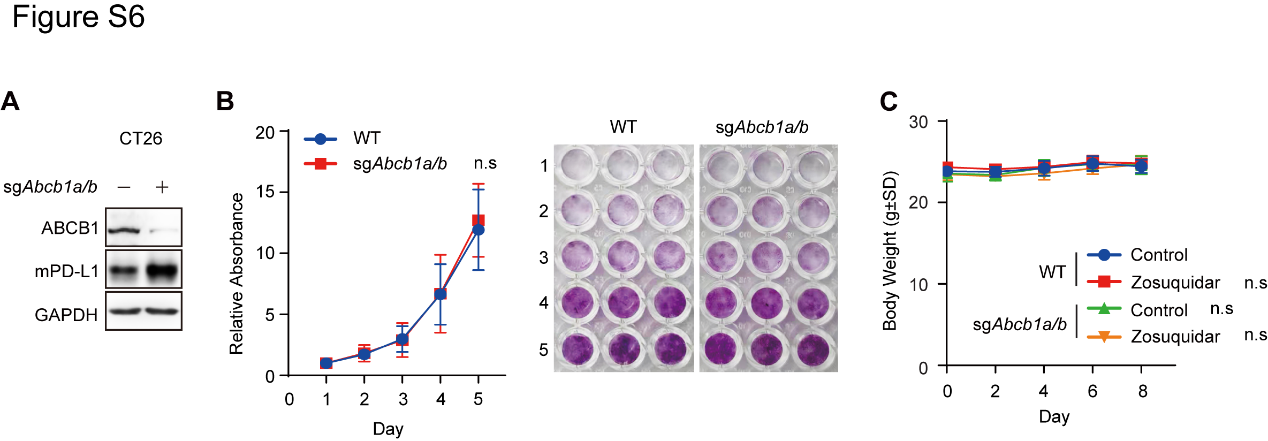


**Figure S6.** Zosuquidar suppressed tumor growth dependent on ABCB1. (A) Western blot of ABCB1 and PD-L1 expression in *Abcb1a/b* knockout CT26 cells. (B) Cell proliferation was detected by SRB assay; representative images were shown on the right. (C) Body weight of each group was measured every two days. Data were presented as the mean ± SD. Unpaired 2-tailed Student’s *t*-test (two groups) and One-way ANOVA with Dunnett’s post hoc test (more than two groups) were used to determine statistical significance of experimental data. n.s, not significantly different.

**Table S1.** Information of anti-cancer drugs library, related to Figure 1

| Number | name | Number | name | Number | name |
| --- | --- | --- | --- | --- | --- |
| 1 | CH5183284 | 102 | PF4136309 | 203 | Nelarabine |
| 2 | Berzosertib | 103 | Poziotinib | 204 | GZD824 Dimesylate |
| 3 | BI847325 | 104 | Salirasib | 205 | MK-2206 dihydrochloride |
| 4 | PF477736 | 105 | AZD2461 | 206 | Alpelisib |
| 5 | Quisinostat | 106 | Tegafur | 207 | Baricitinib |
| 6 | Citarinostat | 107 | BMS777607 | 208 | GSK461364 |
| 7 | GDC-0575 | 108 | Cilengitide | 209 | Nedaplatin |
| 8 | CXD-101 | 109 | MK0752 | 210 | TRX818 |
| 9 | Troglitazone | 110 | L-778123 hydrochloride | 211 | Asciminib |
| 10 | Kevetrin hydrochloride | 111 | Flavopiridol hydrochloride | 212 | GSK2256098 |
| 11 | Combretastatin A4 | 112 | 3-Bromopyruvic acid | 213 | Jaceosidin |
| 12 | ORY1001 | 113 | Histamine 2HCl | 214 | Satraplatin |
| 13 | Berbamine | 114 | Bemcentinib | 215 | Masitinib |
| 14 | Bexarotene | 115 | Amonafide | 216 | Methylisoindigotin |
| 15 | Icaritin | 116 | Briciclib | 217 | Lazertinib |
| 16 | Flupirtine maleate | 117 | INCB057643 | 218 | Mubritinib |
| 17 | Nintedanib Ethanesulfonate Salt | 118 | AZD0156 | 219 | Nifurtimox |
| 18 | Fosbretabulin Disodium | 119 | PF04691502 | 220 | AZD8330 |
| 19 | Retinol | 120 | GDC0810 | 221 | Lonafarnib |
| 20 | Ivosidenib | 121 | AZD3759 | 222 | Ralimetinib Mesylate |
| 21 | S49076 | 122 | Seclidemstat | 223 | AZD-7648 |
| 22 | (Z)-Semaxinib | 123 | Anlotinib | 224 | NXY059 |
| 23 | AMG337 | 124 | LXS196 | 225 | Binimetinib |
| 24 | Dovitinib lactate | 125 | ABBV744 | 226 | RO4987655 |
| 25 | AZD6738 | 126 | Capmatinib | 227 | AZD1208 |
| 26 | Minomustine | 127 | Cediranib | 228 | Serabelisib |
| 27 | Delanzomib | 128 | Galunisertib | 229 | C188-9 |
| 28 | INO1001 | 129 | Navarixin | 230 | Serdemetan |
| 29 | Decitabine | 130 | Neticonazole Hydrochloride | 231 | JI101 |
| 30 | AZD5153 6-Hydroxy-2-naphthoic acid | 131 | Talazoparib | 232 | SCH900776 S-isomer |
| 31 | Isosteviol | 132 | AMG900 | 233 | Apatinib |
| 32 | ORY-1001(trans) | 133 | Galeterone | 234 | Capmatinib 2HCl |
| 33 | Dabrafenib | 134 | Sapitinib | 235 | AZD4547 |
| 34 | Tivantinib | 135 | Pirfenidone | 236 | Volasertib |
| 35 | Sodium Dichloroacetate | 136 | ATB 346 | 237 | Ipatasertib |
| 36 | Licochalcone A | 137 | AZD9496 | 238 | Ulixertinib |
| 37 | Epacadostat | 138 | Ixabepilone | 239 | Alectinib |
| 38 | Infigratinib | 139 | Vesnarinone | 240 | Belvarafenib |
| 39 | Evofosfamide | 140 | Duvelisib | 241 | MGCD-265 analog |
| 40 | Sufugolix | 141 | TGR1202 | 242 | Benzenebutyric acid |
| 41 | Cephalotaxine | 142 | AT13148 | 243 | JNJ38877605 |
| 42 | Bardoxolone | 143 | DCC2036 | 244 | ASP 3026 |
| 43 | KX2-391 | 144 | E7820 | 245 | Brequinar |
| 44 | Acalabrutinib | 145 | CI994 | 246 | SNS-314 Mesylate |
| 45 | LY3200882 | 146 | eFT508 | 247 | MK2461 |
| 46 | Tandutinib | 147 | Ostarine | 248 | Tirapazamine |
| 47 | Motesanib | 148 | Rucaparib Phosphate | 249 | Luminespib |
| 48 | Icotinib | 149 | Vorasidenib | 250 | AST2818 mesylate |
| 49 | Birabresib | 150 | CC-401 Hydrochloride | 251 | Idelalisib |
| 50 | Epothilone B | 151 | PRIMA-1Met | 252 | Cabozantinib hydrochloride (849217-68-1(free base)) |
| 51 | AZD3965 | 152 | Vinorelbine Tartrate | 253 | Ensartinib |
| 52 | Pentostatin | 153 | 4-Hydroxytamoxifen | 254 | NVP-LDE225 |
| 53 | L-(-)-Fucose | 154 | Perillyl alcohol | 255 | NMS-P937 |
| 54 | MK4101 | 155 | Sotrastaurin | 256 | CC115 |
| 55 | DMXAA | 156 | Nimustine Hydrochloride | 257 | Vistusertib |
| 56 | Enzastaurin | 157 | Plinabulin | 258 | Encorafenib |
| 57 | Tepotinib | 158 | Idasanutlin | 259 | SGI-1776 free base |
| 58 | Nazartinib | 159 | Bafetinib | 260 | Homoharringtonine |
| 59 | CCT245737 | 160 | Desmopressin | 261 | TIC10 |
| 60 | 2-Cl-IB-MECA | 161 | Darolutamide | 262 | Telatinib |
| 61 | 1H-Pyrazole-3-acetamide | 162 | LGK974 | 263 | Cobimetinib |
| 62 | CPI-444 | 163 | Embelin | 264 | Linrodostat |
| 63 | GSK690693 | 164 | RGX-104 HCl | 265 | TAS120 |
| 64 | Pipobroman | 165 | Vicriviroc maleate | 266 | Merestinib |
| 65 | CB839 | 166 | Pirarubicin | 267 | Amsacrine |
| 66 | Tazarotene | 167 | Molibresib | 268 | Spebrutinib |
| 67 | ON-01910 | 168 | BLZ945 | 269 | Rubitecan |
| 68 | Calcium Levofolinate | 169 | Elesclomol | 270 | Gedatolisib |
| 69 | Mivebresib | 170 | Canertinib | 271 | (S)-crizotinib |
| 70 | Larotrectinib | 171 | Vismodegib | 272 | Tanespimycin |
| 71 | GSK2879552 2HCl (1401966-69-5(free base)) | 172 | zanubrutinib | 273 | TPX0005 |
| 72 | Entrectinib | 173 | Altiratinib | 274 | AZD5438 |
| 73 | Avadomide | 174 | Gandotinib | 275 | IPI-549 |
| 74 | COH29 | 175 | Amuvatinib | 276 | Topotecan |
| 75 | azd1390 | 176 | Trametinib | 277 | Copanlisib |
| 76 | Hydroxy Camptothecine | 177 | Bromodeoxyuridine | 278 | HSP990 |
| 77 | Niraparib | 178 | RRX-001 | 279 | PND1186 |
| 78 | BLU554 | 179 | Nimorazole | 280 | AZD8055 |
| 79 | Bestatin hydrochloride | 180 | Vactosertib | 281 | Voxtalisib |
| 80 | Gossypol | 181 | Cridanimod | 282 | CH5132799 |
| 81 | Camostat mesilate | 182 | BMS 599626 2HCl (873837-23-1(HCl)) | 283 | SNS032 |
| 82 | PAC1 | 183 | Erastin | 284 | Uprosertib |
| 83 | Relugolix | 184 | CI1040 | 285 | AZD8186 |
| 84 | BMS754807 | 185 | GDC0623 | 286 | GDC0084 |
| 85 | PI3K-IN-2 | 186 | Chlorotrianisene | 287 | Olmutinib |
| 86 | GSK3326595 | 187 | ABC294640 | 288 | AT 7519 hydrochloride salt |
| 87 | PX-478 2HCl | 188 | ZSTK474 | 289 | TP3654 |
| 88 | Berberine | 189 | Doramapimod | 290 | CYC116 |
| 89 | Brivanib Alaninate | 190 | Fruquintinib | 291 | AT7519 |
| 90 | XL413 hydrochloride | 191 | Edoxudine | 292 | Pluripotin |
| 91 | AT9283 | 192 | Roquinimex | 293 | Selonsertib |
| 92 | Calcitriol | 193 | LB100 | 294 | KW2449 |
| 93 | AST-1306 TsOH | 194 | Gemcitabine HCl | 295 | Brigatinib |
| 94 | Efaproxiral Sodium | 195 | AMG208 | 296 | Ribociclib |
| 95 | Fenretinide | 196 | AZD1480 | 297 | PF562271 |
| 96 | OSI 930 | 197 | Avapritinib | 298 | Osimertinib mesylate |
| 97 | Lorlatinib | 198 | Omipalisib | 299 | Capivasertib |
| 98 | Cerdulatinib | 199 | UNBS5162 | 300 | Fludarabine Phosphate |
| 99 | Telotristat Etiprate | 200 | FK866 | 301 | Amlodipine |
| 100 | Mocetinostat | 201 | Irosustat | 302 | Zosuquidar 3HCl |
| 101 | Tamibarotene | 202 | Ravoxertinib |  |  |

**Table S2.** The sequences for siRNA

| **Gene name** | **Primer Sequence (5’ - 3’)** |
| --- | --- |
| *ATG5* | CCUGAACAGAAUCAUCCUUAA |
| *ATG7* | GCCUGCUGAGGAGCUCUCCAU |
| *SQSTM1* | CCUCUGGGCAUUGAAGUUGAU |
| *ABCB1* #1 | CCGAACACAUUGGAAGGAAAU |
| *ABCB1* #2 | CGACAGAAUAGUAACUUGUUU |
| *HDAC6* | CGGUAAUGGAACUCAGCACAU |
| *NBR1* | GCCAGGAACCAAGUUUAUCAA |
| *TOLLIP* | CCAACAAGAUUCCCGUGAAAG |
